# Public Interest in Vitamin C Supplementation During the COVID-19 Pandemic as a Potential Risk for Oxalate Nephrolithiasis

**DOI:** 10.7759/cureus.79452

**Published:** 2025-02-22

**Authors:** Jayson P Kemble, Christine W Liaw, Jamal M Alamiri, Garrett N Ungerer, Aaron M Potretzke, Kevin Koo

**Affiliations:** 1 Urologic Surgery, University of Nebraska Medical Center, Omaha, USA; 2 Urology, Hartford HealthCare, Bridgeport, USA; 3 Urology, Cleveland Clinic, Cleveland, USA; 4 Urology, Mayo Clinic, Rochester, USA

**Keywords:** covid-19, nephrolithiasis, oxalate, pandemic, vitamin c

## Abstract

Introduction: Vitamin C (ascorbic acid) therapy was widely touted as a potential treatment or preventive therapy for COVID-19 despite a lack of supporting evidence. One potential harm of high-dose vitamin C supplementation is increased urinary oxalate, which may increase the risk of hyperoxaluria and oxalate kidney stones. This study aims to evaluate public interest in vitamin C during the COVID-19 pandemic based on online search volume and to characterize variation in vitamin C interest as a potential contributor to kidney stone formation.

Methods: The volume and frequency of online search traffic related to vitamin C and COVID-19 were assessed using the Google Trends platform (Google LLC, Mountain View, CA, USA) between 2018 and 2022. Weekly relative search volumes (RSV), the proportional volume of online searches for a search term, were assessed to compare variations in online interest in vitamin C and COVID-19. The most popular Google search results for vitamin C as a treatment for COVID-19 were assessed for medical accuracy. Statistical analysis was performed with t-tests and linear regression.

Results: Online search volume for vitamin C increased four-fold at the onset of COVID-19 in March 2020. After the initial outbreak, average RSV for vitamin C remained significantly elevated compared to pre-COVID-19 levels (37.7 vs. 25.1, p<0.001). Weekly RSV for vitamin C increased steadily during the study period (R2=0.59, p<0.001). The peak in online interest in vitamin C corresponded to increased online search volume during three global COVID-19 surges. Among the most popular results for COVID-19-related vitamin C queries, 30% inaccurately suggested that vitamin C had potential benefits in treating COVID-19. None of these search results discussed the potential increased risk of kidney stones with vitamin C supplementation.

Conclusion: Online public interest in vitamin C supplementation increased and remained elevated during the COVID-19 pandemic. These findings have implications for increased risk of hyperoxaluria and oxalate stones due to vitamin C supplementation. Kidney stone patients should be counseled that excess vitamin C intake is associated with increased urinary oxalate and incident stone formation.

## Introduction

At the start of the COVID-19 pandemic, there was widespread public interest in finding preventive therapy and treatment options for COVID-19, with many individuals looking into complementary and alternative medicine. In the first six weeks of public awareness of COVID-19 in the United States, between February 23 and April 5, 2020, there was a 44% increase in sales of vitamins and other dietary supplements, totaling $435 million [1]. Among the most popular dietary supplements among U.S. consumers has been vitamin C (ascorbic acid), which is commonly believed to treat and protect against viral illnesses despite a lack of evidence in the scientific literature to support this claim [2]. Specifically, the U.S. National Institutes of Health has concluded that there is insufficient evidence to recommend for or against the use of vitamin C in the treatment of COVID-19 [3]. In contrast, the proliferation of vitamin C-based powders and tablets, which are largely unregulated by the U.S. Food and Drug Administration, underscores the sustained consumer interest in vitamin C.

Public interest in high-dose vitamin C supplementation is of potential concern to urologists and nephrologists because vitamin C is metabolized to oxalate and may result in hyperoxaluria, a known risk factor for kidney stone development [4,5]. A 1 gm dose of vitamin C, which is contained in a typical single dose of “immune health” products like Emergen-C, increases urinary oxalate up to 60% [6,7]. In a population-based prospective cohort study, adult men who took daily vitamin C supplements were twice as likely to develop kidney stones, whereas multivitamin use was not associated with increased kidney stone risk [8]. However, this association between vitamin C hyper-supplementation and lithogenic risk may not be commonly known or discussed. As variants of COVID-19 continue to emerge and cause recurrent surges in disease, sustained public interest in vitamin C during the pandemic has both public health and urological relevance but has not been characterized.

This study aims to evaluate public interest in vitamin C before and since the COVID-19 pandemic and to characterize temporal variation in vitamin C interest as a potential contributor to higher rates of oxalate kidney stone formation. This study was previously presented as a meeting abstract at the 2022 North Central Section of the American Urological Association Annual Meeting on August 30, 2022.

## Materials and methods

The Google Trends platform (Google LLC, Mountain View, CA, USA) is a public database of internet search traffic on Google, the most popular internet search engine by volume. This platform is a reliable source of both public interest and health trends, and it has been demonstrated to accurately predict regional COVID-19 outbreaks based on surges in search traffic for COVID-19 symptoms and treatments [9]. Google Trends was queried to record the relative search volume (RSV) for user searches in the U.S. related to vitamin C and COVID-19 from January 2018 to August 2022. The RSV is reported as a proportional value, ranging from 0-100, with a value of 100 representing the peak search volume and popularity over the study period.

We queried “vitamin C” and “COVID-19” and variations of these terms, independently and in combination, to track search volumes over the study period. We queried “vitamin E” to assess RSV changes for an unrelated term during the study period. We also queried “influenza” and “flu” alongside the primary search terms to assess the seasonal periodicity of searches. Google Trends provides the option to compare two terms to each other, which was used when RSV values between terms were relatively close.

To assess the medical accuracy of online information about the use of vitamin C as a preventive or therapeutic treatment for COVID-19, we performed a Google search for “vitamin C” and “COVID-19” and analyzed the websites returned in the first page of search results for clinical accuracy and references to scientific evidence. All queries were performed in a login-neutral, cache-cleared manner to minimize algorithmic bias based on search history or user-related factors.

Statistical analysis was performed with the Student's t-tests and linear regression. Linear regression excluded the three-month peak surrounding the initial COVID-19 outbreak to enhance modeling of the overall trend in public online interest in vitamin C. Because Google Trends data are adjusted to fit the 0-100 scale, inclusion of the very large spike in RSV during the initial COVID-19 outbreak would have dampened RSV for all other periods as relative noise, so exclusion of the peak allowed for amplification of relevant signals to permit characterization. An ethics review was not required for this analysis of public, non-patient data.

## Results

**Figure 1:**
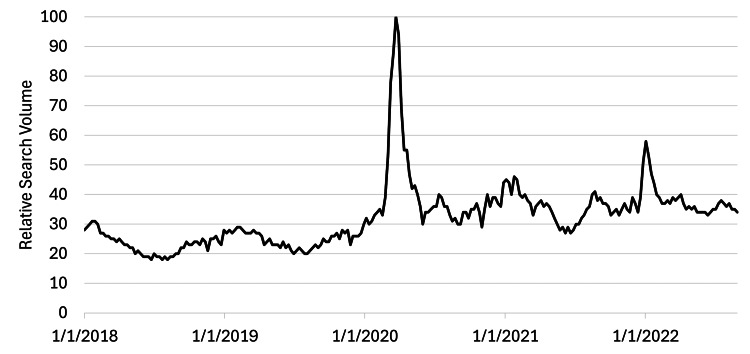
Online search interest in vitamin C Searches for vitamin C during the COVID-19 outbreak in March 2020 were four times higher than any previous peak. Subsequent outbreaks also led to diminished surges in online interest, about half the volume of the initial peak in early 2020.

Online searches for vitamin C peaked in March 2020, at the start of the COVID-19 pandemic, increasing sharply four-fold from baseline starting the week of March 8, 2020 (Figure 1). After the initial outbreak, searches for vitamin C remained consistently elevated compared to pre-COVID-19 levels. Comparing August 2018-2019 to August 2021-2022, baseline interest in vitamin C increased by 50%, from a yearly RSV average of 25.1 to 37.7 (p<0.001). In contrast, interest in vitamin E showed no appreciable change, from a yearly RSV average of 10.0 to 10.3 (p>0.05) during the same periods.

**Figure 2:**
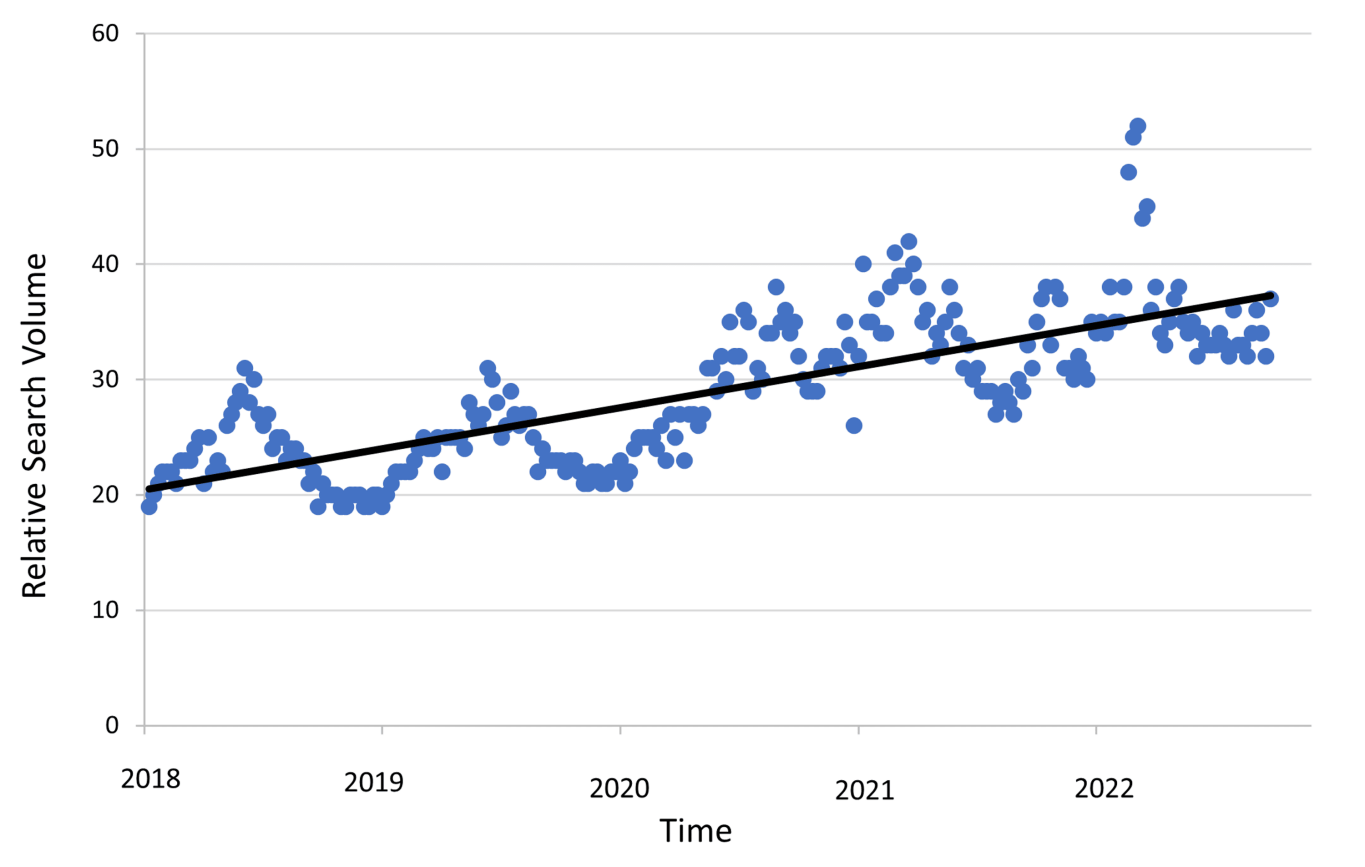
Weekly variation in online interest in vitamin C Linear regression for vitamin C searches by week, excluding the three-month peak surrounding the initial COVID-19 outbreak (R^2^=0.59; p<0.001).

A linear regression model of online search interest in vitamin C before and after the initial surge in March 2020 confirmed rising overall interest, with periodic peaks likely driven by seasonal flu and COVID-19 variant outbreaks (R^2^=0.59; p<0.001) (Figure 2). 

**Figure 3:**
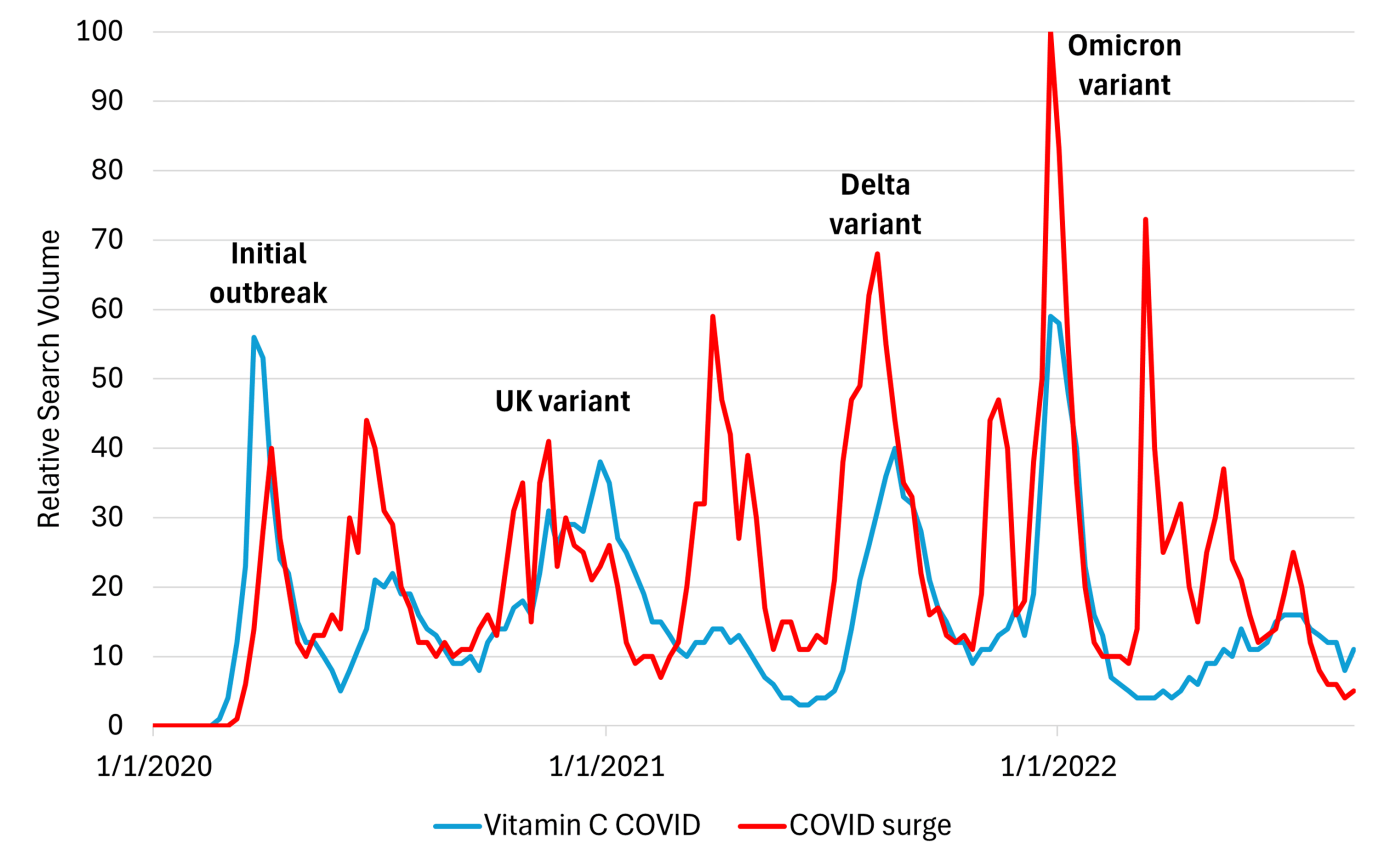
Comparative analysis of online search interest between vitamin C and COVID-19 surges Similar spikes in search patterns emerged during the initial outbreak in March 2020 and subsequent surges of COVID-19 variants (UK variant in December 2020, delta variant in July 2021, and omicron variant in early 2022).

To compare volumes between searches for COVID-19 and vitamin C, we used “COVID surge” as a proxy term because the search volume for “COVID” far exceeded any other search term and would preclude meaningful comparisons between relative search volumes. The term “COVID surge” was confirmed to follow the same patterns in search volume over time as “COVID” alone. Figure 3 compares online search interest between vitamin C and COVID-19 over the study period. The popularity of searches for both terms was found to follow similar patterns: when searches for COVID surge were trending, there was frequently a corresponding increase in searches for vitamin C. These peaks corresponded to notable global surges or waves in COVID-19: the initial outbreak in March 2020, the UK variant in December 2020, the delta variant in July 2021, and the omicron variant surge in early 2022. Notably, both search terms peaked higher in 2022 than they did in 2020, suggesting not only sustained online interest in COVID-19-related vitamin C over time but also relative increases in vitamin C searches corresponding to surges of COVID-19 cases. 

To assess interest in COVID-19-related vitamin C and the potential risk of kidney stones, we compared search terms related to COVID-19 and kidney stones. There was no identifiable interaction or pattern in search volumes for the two topics, with interest in both terms remaining relatively stable during the study period. 

Finally, we assessed the most popular search results for general online searches for vitamin C and COVID-19 to determine whether users who searched for these terms would encounter medically accurate information about vitamin C as a preventive or therapeutic treatment for COVID-19. The top 10 website results were medical websites including patient health information published by scientific publications and academic institutions. Vitamin C was stated to have potential benefits in treating COVID-19 on 30% of these websites. None of the search results explicitly recommended against using vitamin C in COVID-19. Finally, none of the websites discussed any potential harms of hyper-supplementation of vitamin C or any increased risk of kidney stones.

## Discussion

In the early days of the COVID-19 pandemic, the public desire for home remedies that could prevent or treat the condition led to increased interest in vitamins, micronutrients, naturopathic supplements, and other remedies, despite no evidence of benefit. Vitamin C in particular gained substantial popularity due to widely held beliefs that it could treat or cure viral illness. In our assessment of public interest in vitamin C for COVID-19, online search volume increased sharply in March 2020, and despite the absence of scientific evidence or federal recommendations recommending vitamin C to prevent or treat COVID-19, baseline interest two years into the pandemic remained significantly elevated. Notably, the websites most frequently encountered by patients conducting these searches never discussed hyperoxaluria or the potential risk of oxalate stone formation.

Public interest in vitamin C therapy has traditionally had seasonal peaks during yearly flu seasons. With the onset of the COVID-19 pandemic vitamin C interest spiked but did not return to baseline levels, with interest remaining 50% higher than pre-COVID-19 levels, suggesting continued interest in vitamin C even when COVID-19-specific therapies became widely available, including vaccines, monoclonal antibodies, and oral medications. In contrast, search volumes for vitamin E, which we assessed as an unrelated term to characterize internet search volumes overall, did not change appreciably during the study period, suggesting that the trends we identified for vitamin C reflect actual increases in public interest. The highest spikes of new U.S. COVID-19 cases occurred in the winter of 2020, late summer of 2021, and the beginning of 2022, which coincided with peaks in vitamin C interest [10]. This heightened interest could be due to several factors, including public uncertainty and anxiety surrounding variant COVID-19 strains and variable levels of public trust in traditional medicine. Notably, excess vitamin C intake is associated with urinary saturation of oxalate, so increased interest in vitamin C hyper-supplementation could increase the incidence of oxalate stone formation [5].

Despite the known association between vitamin C intake and oxaluria, none of the Google search results most commonly encountered by the public included any discussion of kidney stones as a risk of vitamin C supplementation, increasing the concern that individuals may unknowingly increase their risk of incident kidney stones. In addition, some websites included misleading or medically inaccurate information about vitamin C use for COVID-19. For instance, one major news outlet reported the following as expert guidance: “Vitamin C, we know it's working well. There have been some studies on COVID-19 where vitamin C has shown to have very good benefits in the outcome of an illness” [11]. There were no studies referenced in this article, and we were unable to identify any studies in the medical literature that supported this claim. This exemplifies the challenge that patients may encounter in deciphering medical information from conflicting online sources. 

Limitations

A limitation of our study was that some search terms were not able to be directly compared to each other due to large differences in RSV that would reduce the sensitivity of detecting changes in search patterns. We were also unable to directly correlate increases in vitamin C interest with rates of vitamin C consumption or incident nephrolithiasis, which was beyond the scope of the study. We acknowledge that many factors may have influenced public interest in vitamin C during the study period, including changes in the media environment, celebrity endorsements, or marketing by dietary supplement manufacturers during the pandemic. Although we were unable to assess or control for these factors directly, our findings are likely to reflect overall trends in vitamin C supplementation, which may be of interest to clinicians, researchers, and public health leaders. Publicly available data on the incidence of kidney stone treatments in large national databases such as the National Surgical Quality Improvement Program were updated only to 2020 at the time of the study and would not reflect more recent trends. Furthermore, vitamin C supplementation would not be expected to be the primary or sole contributor to potentially increased rates of nephrolithiasis during COVID-19. Further studies using systematic surveys of nutrition and diet, such as the National Health and Nutrition Examination Survey, may support and extend our exploratory findings.

Despite these limitations, this study has important implications. First, the COVID-19 pandemic is likely to have resulted in important and potentially sustained changes in the way patients use dietary supplements, both for perceived antiviral properties and health benefits, though these benefits have not been supported by evidence [3]. The commercial market for dietary supplements to treat and prevent kidney stones is vast, but the majority of these products contain ingredients for which no scientific evidence supports claims of use in stone disease [12]. 

Second, these findings support accumulating evidence that demonstrates inconsistent reliability of patient information about kidney stones on the internet. Social media sources, such as YouTube, have for some patients become a default search engine for health information. In contrast to typical search engines, which may present websites from reputable sources of consumer health information like hospitals or academic centers, social media platforms present primarily user-generated content, which for kidney stone information often lacks quality and accuracy and has been found to be inconsistent with evidence-based clinical guidelines for other urological conditions like benign prostatic hyperplasia [13]. The need for reliable patient education about kidney stones is increasingly important, as recent surveys have estimated that the 12-month incidence of kidney stones in the US is now over 2% in adults over the age of 20 [14]. 

Third, urologists should be aware of the sustained increase in public interest in vitamin C supplementation and its potential impact on oxalate stone formation. The findings merit continued study of the incidence of nephrolithiasis during and after the COVID-19 pandemic, which may be attributable to pandemic-related changes in vitamin C use, as well as other important factors like diet, nutritional supplement use, and delayed kidney stone care. 

Finally, studies of internet search behavior can help clinicians, public health officials, and the news media understand changes in public interest in health conditions. Our findings of sustained public interest in vitamin C during and following a public health crisis may help these parties work together to contextualize this interest in a scientifically responsible way and to combat potential misinformation that may have unintended health consequences, such as the increased risk of oxalate stone formation. Findings from these studies should also be interpreted carefully in the context of online privacy, data availability, and the potential impact of algorithmic bias.

## Conclusions

Online public interest in vitamin C supplementation increased sharply during the COVID-19 pandemic and remained elevated, despite a lack of evidence supporting its use. More widespread use of high-dose vitamin C supplementation may have clinical implications for increased oxalate stone formation. Clinicians should counsel patients that excess vitamin C intake is associated with increased urinary oxalate and may increase the risk for incident kidney stone formation.

## References

[1] Lordan R (2021). Dietary supplements and nutraceuticals market growth during the coronavirus pandemic — Implications for consumers and regulatory oversight. PharmaNutrition.

[2] Cerullo G, Negro M, Parimbelli M (2020). The long history of vitamin C: from prevention of the common cold to potential aid in the treatment of COVID-19. Front Immunol.

[3] (2022). Coronavirus Disease 2019 (COVID-19) Treatment Guidelines. https://www.covid19treatmentguidelines.nih.gov.

[4] Crivelli JJ, Mitchell T, Knight J, Wood KD, Assimos DG, Holmes RP, Fargue S (2020). Contribution of dietary oxalate and oxalate precursors to urinary oxalate excretion. Nutrients.

[5] Khusid JA, Atallah WM, Kyprianou N, Gupta M (2020). What stone-formers should know about vitamin C and D supplementation in the COVID-19 era. Eur Urol Open Sci.

[6] (2022). Emergen-C Dietary Supplements for Everyday Wellness. https://www.emergenc.com/.

[7] Baxmann AC, De O G Mendonça C, Heilberg IP (2003). Effect of vitamin C supplements on urinary oxalate and pH in calcium stone-forming patients. Kidney Int.

[8] Thomas LD, Elinder CG, Tiselius HG, Wolk A, Akesson A (2013). Ascorbic acid supplements and kidney stone incidence among men: a prospective study. JAMA Intern Med.

[9] Venkatesh U, Gandhi PA (2020). Prediction of COVID-19 outbreaks using Google Trends in India: a retrospective analysis. Healthc Inform Res.

[10] (2022). United States COVID - Coronavirus Statistics - Worldometer. https://www.worldometers.info/coronavirus/country/us/.

[11] Wilson M (2022). Studies suggest 4 vitamins to lower risk of severe cases of COVID-19 | FOX 26 Houston. https://www.fox26houston.com/news/studies-suggest-4-vitamins-to-prevent-severe-cases-of-covid-19.

[12] Koo K, Aro T, Matlaga BR (2020). Buyer beware: evidence-based evaluation of dietary supplements for nephrolithiasis. J Endourol.

[13] Huang MM, Winoker JS, Matlaga BR, Allaf ME, Koo K (2021). Evidence-based analysis of online consumer information about prostate artery embolization for benign prostatic hyperplasia. Prostate Cancer Prostatic Dis.

[14] Hill AJ, Basourakos SP, Lewicki P (2022). Incidence of kidney stones in the United States: the continuous National Health and Nutrition Examination Survey. J Urol.

